# Recent advance of herbal medicines in cancer- a molecular approach

**DOI:** 10.1016/j.heliyon.2023.e13684

**Published:** 2023-02-14

**Authors:** Mohammad Ali, Shahid Ud Din Wani, Md Salahuddin, Manjula S.N., Mruthunjaya K, Tathagata Dey, Mohammed Iqbal Zargar, Jagadeesh Singh

**Affiliations:** a Department of Pharmacy Practice, East Point College of Pharmacy, Bangalore, 560049, India; b Department of Pharmaceutical Sciences, School of Applied Sciences and Technology, University of Kashmir, Srinagar, 190006, India; c Department of Pharmaceutical Chemistry, Al-Ameen College of Pharmacy, Bangalore, 560027, India; d Department of Pharmacology, JSS College of Pharmacy Mysuru, JSS Academy of Higher Education and Research, Mysuru, 570004, India; e Department of Pharmacognosy, JSS College of Pharmacy Mysuru, JSS Academy of Higher Education and Research, Mysuru, 570004, India; f Department of Pharmaceutical Chemistry, East Point College of Pharmacy, Bangalore, 560049, India; g Department of Pharmacognosy, East Point College of Pharmacy, Bangalore, 560049, India

**Keywords:** Anticancer, Phytochemicals, Pharmacology, Autophagy, Apoptosis, Molecular mechanism

## Abstract

Bioactive compounds are crucial for an extensive range of therapeutic uses, and some exhibit anticancer activity. Scientists advocate that phytochemicals modulate autophagy and apoptosis, involved in the underlying pathobiology of cancer development and regulation. The pharmacological aiming of the autophagy-apoptosis signaling pathway using phytocompounds hence offers an auspicious method that is complementary to conventional cancer chemotherapy. The current review aims to explore the molecular level of the autophagic-apoptotic pathway to know its implication in the pathobiology of cancer and explore the essential cellular process as a druggable anticancer target and therapeutic emergence of naturally derived phytocompound-based anticancer agents. The data in the review were collected from scientific databases such as Google search, Web of Science, PubMed, Scopus, Medline, and Clinical Trials. With a broad outlook, we investigated their cutting-edge scientifically revealed and/or searched pharmacologic effects, a novel mechanism of action, and molecular signaling pathway of phytochemicals in cancer therapy. In this review, the evidence is focused on molecular pharmacology, specifically caspase, Nrf2, NF-kB, autophagic-apoptotic pathway, and several mechanisms to understand their role in cancer biology.

## Abbreviations

**EGCG**: epigallocatechin gallate
**TCM**: Traditional Chinese Medicines
**SHH**: Sonic Hedgehog Homology
**NSCLC**: Non-Small-Cell Lung Cancer
**PAM + V**: Pan-Asian drug and vitamins
**UHCC**: Unresectable Hepatocellular Carcinoma
**CONSORT**: Consolidated standards of reporting trials
**PAS**: Phagophore assembly sites
**PI3K**: Phosphatidylinositol 3-phosphate
**ER**: Endoplasmic reticulum
**ROS**: Reactive oxygen species
**AMPK**: AMP-stimulated protein kinase
**ULK1**: unc-51-like autophagy activating kinase-1
**TNF**: Tumour necrosis factor
**FasL**: Fas ligand
**FADD**: Fas-related death domain protein
**BAK**: Bcl-2 antagonist killer 1
**Bax**: Bcl-2-associated X protein
**BID**: BH3 interacting-domain death agonist
**SMAC**: Second mitochondrial-derived activator of caspases
**FADD**: Fas-associated death domain
**DISC**: Death inducing signaling complex
**PARP**: Poly (ADP-ribose polymerase
**mTOR**: Mammalian target of rapamycin
**AMPK**: AMP-activated protein kinase
**EGFR**: Epidermal growth factor receptor
**STAT3**: Signal transducer and activator of transcription 3
**HER2**: Human epidermal growth factor receptor 2
**DDR**: DNA damage response
**CHD**: Coronary heart disease
**MAPK**: Mitogen-activated protein kinase
**Nrf2**: Nuclear factor erythroid 2–related factor 2
**ARE**: Antioxidant Response Element
**PKC**: Protein kinase C
**VEGF**: Vascular endothelial growth factor
**FGF**: Fibroblast growth factor
**MMPs**: Matrix metalloproteinases
**CAFs**: Cancer-related fibroblasts
**TAMs**: Tumour-related macrophages;
**CSCs**: Cancer stem cells
**PKG**: Protein kinase G
**iNOS**: Inducible nitric oxide synthase
**EMT**: Epithelial-mesenchymal transition
**MIC**: Minimum Inhibitory Concentration
**NDDSs**: Nanoparticle-based drug delivery systems
**MDR**: Multidrug resistance

## 1 Introduction

Globally, cancer is the most serious health complication suffering by a human being, with an estimated new cases total of 19.3 million and nearly 10.0 million cancer-related deaths in 2020 [1,2]. In 2021, the USA is anticipated to have 18,98,160 new cases and 6,08,570 deaths due to cancer [3]. Herbal remedy has been used as anticancer drug for a decade-long period, display anti-inflammatory effects, and contain plentiful components that possess anticancer effect that showed direct cytotoxic impacts and indirect regulation in tumour micro-environment, cancer immunity, and progress chemotherapy [4,5]. For example, PNAS stated that phytoconstituents i.e. epigallocatechin gallate (EGCG) directing Laminin receptor (Lam67R) display auspicious efficacy in treating prostate cancer [6]. Literature demonstrated that ginsenoside Rh2 prevents *P*-glycoprotein (*P*-gp) action to converse multi-drug resistance [7]. Earlier literature reported that curcumin causes autophagy to augment apoptosis [8]. Researchers studied that berberine possibly suppresses cancer development and brings safe for cancer sufferers [9]. Herbal Medicine displayed that shikonin induces synergistic impacts on the use of anticancer drugs [10]. However, the anticancer targets of these compounds are still unclear, and this is the foremost impediment to the use and progress of natural medicine. The current review in natural medicines and cancer emphases on brief investigational outcomes and conclusions. Numerous phytochemicals are obtained from herbal remedies i.e. curcumin, ellagic acid, EGCG, berberine, artemisinins, ginseng, gallic acid, artesunate, etc. were recognized to develop anticancer properties, i.e. antiproliferative, antiangiogenic proapoptotic, antimetastatic impacts, and control of autophagy, the reverse of drugs resistance, immunity stability, and development of chemotherapy by *in-vitro* as well as *in-vivo*. Nonetheless, autophagy and programmed cell death introduction and/or prevention are enormously multifaceted processes that require in-depth investigation. However, strong consideration of the interplay between apoptosis and autophagy will allow for the development of novel anti-cancer treatment plans. In the current review, we investigate the molecular mechanism of actions of autophagy and apoptosis in malignancy. Assumed the importance of natural components in chemotherapy, we confer a variety of phytochemical constituents that may control autophagy and apoptosis-linked signaling pathways, to improve cancer chemotherapy outcomes [11]. Chemotherapy resistance develops in cancer-infected cells by evading some potential apoptosis mechanisms for example attenuation proapoptotic signals, intensification antiapoptotic signals, and defective apoptosis instigation and application. Despite this, the functional rapport is complex between the apoptosis and autophagy, but recently reported its free from the complexity in the molecular mechanism. Thus, modulating significant aspects in the autophagic and apoptotic pathways could be a unique treatment approach for improving cumulative cancer treatment efficacy [12].

Phytochemicals and their derivatives are capable to enhance therapeutic efficacy in cancer patients and reduce side effects. A number of these phytoconstituents are naturally occurring biologically active compounds with significant antitumor effects. Phytochemical compounds regularly act by controlling molecular signaling pathways that are implicated in the development of cancer. The particular mechanisms such as augmenting antioxidant status, carcinogen inactivation, inhibiting proliferation, initiation of cell cycle arrest and apoptosis, and regulation of the immune system [13].

Traditional herbal medicines have investigated and revealed a large number of anti-breast cancer agents, though many of their mechanisms of action are still unknown. These TCM herbs with anti-breast cancer properties have been divided into six groups: alkaloids [14], coumarins [15,16], polyphenols and flavonoids [17,18], terpenoids [19], quinone [20], and artesunate [21]. Few of them, i.e. chemical structures of curcumin and artemisinin well-known. For a decade the components in these groups have been considered healthy food. Nevertheless, preclinical as well as clinical studies are still suggested for regular human use and/or precise clinical uses [22]. Cancer is produced by the uncontrolled proliferation and development of unhealthy cells, that can cause death. The utmost effective cancer treatments inhibit tumour development and prevent metastasis. State-of-the-art medical technologies and cutting-edge cancer therapies, i.e. cancer chemotherapy, radiotherapy, and immunotherapy, have resulted in successful clinical outcomes in various types of cancer. The immune defense system, which constantly regulates the body for pathogens and incursive antigens, also plays a vital role in the tumour micro-environment, which contributes to cancer heterogeneity. Elimination, equilibrium, and escape are the three stages of immune excision that allow tumour development. Initially, cancer cells avert the body's immune defense system from removing new cancer cells; after that, they accomplish equipoise with the immune cells; and finally, they escape immune investigation. Effective immune treatments in cancer must be able to deregulate immune cells at all three steps and increase the strength of cancer-fighting cells. T cells, e.g., that usually recognize as well as slaughter cancer-infected cells, could become bushed and debilitated, as proved using the addition of an apoptotic indicator (PD-1) [23]. An effective routine cancer treatment also desires to diminish the healthy cells toxicity, as numerous cancer patients reported in cytotoxic therapies but recover toxic effects i.e. cachexia, body weight loss, tiredness, and cytokine storm. Hence, there is an increasing concentration in supplementing common cancer treatments with herbal and nutritional ingredients to progress the QoL of the patients throughout cancer chemotherapy [24]. Globally nutritional immunology is also growing more interest and reported its feat with immune treatment. Herbal remedies and nutritional products are also revealed in in-vitro to influence immune cells and to progress the immune system to defend against many circumstances, i.e. inflammatory diseases and cancer [[25], [26], [27]].

In the present review, the following keywords were used to conduct a literature search in peer-reviewed and clinical study databases, including PubMed, WoS, Medline, Scopus, and Clinical Trials: cancer, tumour, neoplasm, Chinese herbs, and herbal remedy. The pharmacological impacts, a novel mechanism of action, related clinical research, and novel uses in combination treatment of the common components induced by herbal remedies were constantly reviewed to offer new ideas into the critical path ahead.

## 2 Methods

PubMed, Scopus, Web of Science, Google Scholar, and Google search were used to collect data from published articles and related methodological aids of various molecular-based mechanisms of phytochemical constituents in autophagy, apoptosis modulation, and cancer deterrence. Apoptosis, autophagy, phytoconstituents, cancer, solid tumours and lymphomas, immunology, and perspectives on autophagy in chemotherapy were all searched for. All of the figures in this review were created with Adobe Illustrator software.

## 3 Results

### 3.1 Clinical applications of herbal remedy with anti-cancer effects

A variety of clinical studies have revealed that a range of anti-cancer properties from numerous herbal remedies can be explored. Globally, there are classified the clinical use of a wide variety of herbal remedies according to their suppressive impact on precise types of cancer (Name of herbal remedies are not mentioned).

#### 3.1.1 For breast cancer

Though the precise activity of vitamins in the deterrence of breast cancer has not been recognized, some anti-cancer properties have been exposed to in-vitro studies [[28], [29], [30]]. Conducted a randomized controlled trial with 2972 breast cancer patients and consumed either 200 mg preparation of vitamin-A (Fenretinide)/day or no treatment. At 8.1 years post-treatment, it has been found that a significant decrease in the repetition of local breast cancer in pre-menopausal women. However, no significant difference has been found in total survival time [31]. Interestingly, other studies have revealed that long-term acceptance of vitamin E indicates to have an undesirable impact on breast cancer patients [32,33]. Presently, their act looks to induce indigestion in cancer patients suffering from an associated disease, furthermore, patients consuming healthy food [34].

Phytoestrogens are divided into hydrophilic isoflavones and lipophilic lignans. Isoflavones are available in high quantities in soybeans, and polyphenols are available in fruit and vegetables [35,36]. So far among six associated human studies conducted, in those only one surmised that isoflavone was related to an abridged hazard of breast cancer [37]. Phytoestrogens obtained from soybeans are generally administered for the treatment of postmenopausal women with breast cancer who were undergoing the treatment of tamoxifen. The major phytoconstituents such as isoflavones genistein and daidzein of soybean plant extracts, which are similar structurally to 17β*-*estradiol and can show poor estrogenic impacts [38]. Though, there is no proof to assist the agreement of utilization of phytoestrogens either for the treatment of breast cancer or for relieving the symptoms of climacteric [39]. Explorations of traditional Chinese medicines (TCM) have exposed some anti-breast cancer drugs, while a maximum of their mechanisms have not so far been clarified. The TCM such as alkaloids [40,41], coumarins [42,43], flavonoids and polyphenols [44,45], terpenoids [46], quinone [47] and artesunate [48] have anti-breast cancer activities.

#### 3.1.2 For lung cancer

Globally, the most-deadly complication is lung cancer, moreover, metastasis of cancers usually occurs in the lung compared to other organs. Existing anti-cancer drug regimens frequently have inadequate survival benefits because of the high toxicity [49,50] of the numerous anti-cancer drugs, i.e. docetaxel, etoposide, gemcitabine, paclitaxel, and vinorelbine. The earlier study advised that herbal remedies and their phytoconstituents that look to have fewer side effects may offer an interesting plan for the management of lung cancer. Traditionally, herbal plants including *Morus alba*, *Perilla frutescens*, *Tussilago farfara*, etc., have been used for the treatment of lung cancer [51].

Medically, the percentage of cancer patients who practice herbal remedies as adjuvant remedies along with conventional (for example chemotherapy) therapy for lung cancer is as much as 77% [52]. Mostly herbal remedies are utilized to augment anticancer impact and decrease toxic effects in lung cancer treatment [53]. Though, it is crucial to document that some herbs might have toxic impacts or decrease the impact of conventional therapy, and the principal validation for utilization of natural remedies remains practical to prove [54].

#### 3.1.3 For pancreatic cancer

Smoothened (SMO), which is essential to the sonic hedgehog homolog (SHH) signaling pathway, has been revealed to play a crucial role in the cellular behavior of cancer stem cells [55]. The de-regulation of SHH was believed as a vital aspect that can continue the development of pancreatic cancer [56]. The SMO antagonists including GDC-0449, PF-04449913, etc are presently assessing for pancreatic cancer treatment [57]. Steroidal alkaloids such as cyclopamine isolated from Veratrum californicum, can effectively avert SHH signaling through straight binding to the 7-helix bundle of the SMO protein [58]. It desires to be documented that cyclopamine not only can deteriorate the recruitment of bone marrow precursor cells (BMPCs) into cancer cells but also can decline the growth of the blood vessels of tumour [59]. Results advocate that this phytocompound derived from TCM must be effectively described in the future for effective SMO-selective anti-cancer agents.

### 3.2 Application of herbal supplements as adjuvants in conventional anti-cancer treatment

**Fig. 1:**
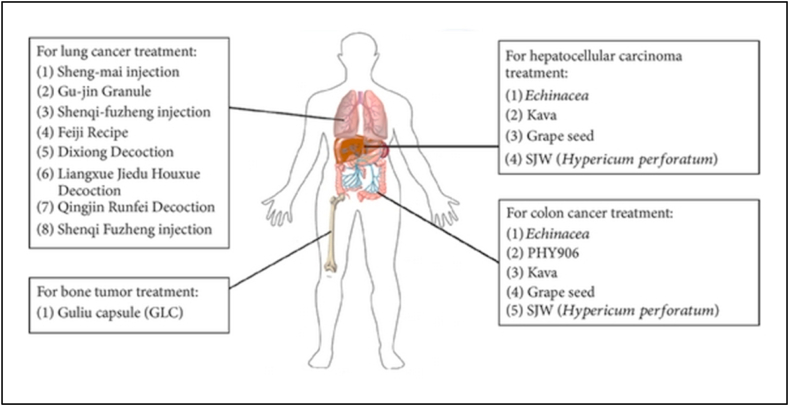
Herbal formulations, such as extracts or formulations, are being studied as supplement therapies for chemotherapy and/or radiotherapy against a variety of cancers. Reproduced with permission from Ref. [82] which was published under a CC BY license.

To enhance the anticancer treatment and decrease toxic effects various herbal remedies are being used in combination with chemotherapy (Fig. 1) as adjuvants to conventional therapy by numerous Western doctors.

Hence, day by day the requirements are augmented by consultation and coordination with doctors and other health care providers. The above segment summaries prove the application of natural herbal remedies as adjuvants to conventional drug-based, chemo-and/or radiotherapy management in cancer therapy. In contrast, this statement also outlines the challenges for the clinical application of these herbal remedies.

#### 3.2.1 Commonly practice of herbal remedies as adjuvant therapy in chemotherapy and/or radiotherapy

In the above-mentioned adjuvant anti-cancer treatment research, herbal medicine was used as a combination treatment with conventional cancer treatment, to augment treatment benefit and QoL while decreasing toxic effects. Between 29% and 99% of racial cancer-infected patients in Asia [60,61] and 26%–47% of them in America [62,63] are stated to have used herbal remedies for cancer treatment. Even though numerous herbal remedies have been discovered to use as a supplement in chemotherapy and radiotherapy, and various clinical trials have been proposed, mostly in China and other countries, they are rarely quoted in PubMed. Many systematic reviews herbal medicines in clinical trials have provided many systematic reviews of herbal remedies in clinical trials, primarily as adjunct therapies to decrease illnesses and the toxic impacts of chemotherapy and/or radiotherapy. Numerous herbal remedies have been used for centuries [[64], [65], [66]].

Curcumin [67,68], Ginseng [69,70], TJ-41 [71,72], PHY906 [73,74], Huachansu [75,76], and Kanglaite [77] are generally consumed by cancer-infected patients for cancer treatment and decrease the noxious effects caused by chemotherapy and radiotherapy. According to pre-clinical and clinical reports, these herbal remedies may have many benefits in terms of tumour suppression by augmenting the sensitivity of chemotherapeutics and/or radiotherapeutics, increasing the action of the body's immune defensive system, and enabling the tissue/physiology injury caused by chemotherapy and/or radiotherapy. Nonetheless, many studies have been published to date to reduce toxic effects during or after chemotherapy and radiotherapy [78,79]. Reported that the protection of hepatic function can decrease cancer-associated complications i.e. fatigue and pain, progress RTI, and GI toxic effects [80]. Suggested valuable indicators for the progression of future herbal medicines as cancer adjunct treatments [81].

#### 3.2.2 For lung cancer

In a randomized controlled trial (RCT) with 63 patients with non-small-cell lung cancer (NSCLC), Sheng-mai Injection (Ya'an Sanjiu Pharmaceutical Co., China) and Gu-jin Granule (Jiangyin Tianjiang Pharmaceutical Co., China) were stated to enhance median survival time (P=0.014) and response rate rise up to 48.5% (16/33), compared to control (untreated) (32.2% = 9/28) with the P=0.0373, whereas entire test clusters were given the treatment of a combination of navelbine and cisplatin (NP) [83].

Reported in one more clinical trial of Shenqifuzheng injection (Lizhu Co., China) the herbal injection significantly enhanced the QoL and response rate of enrolled 232 NSCLC patients, which was evaluated by applying the QoL scale of European Organization for Research on Cancer therapy (QLQ-C30) [84]. Moreover, Feiji Recipe therapy was also found to improve therapeutic efficiency and decrease the toxic impacts of chemotherapy in another the randomized controlled trial (RCT) [85], as revealed with a rise on scores in role, social and economic status (P<0.05orP<0.01), also based on QLQ-C30 questionnaire [86]. Previously, authors [87] applied a clinical trial method to scrutinize the efficacy of TMC on enhancing QoL of postoperative NSCLC patients. In this evidently expressed the design and protocol for a placebo-controlled, double-blinded RCT, where they were able to successfully offer the prove for the sake of the efficiency of chemotherapy combined with TCM in ameliorate QOL of postoperative NSCLC patients. The outcome was anticipated to offer support for consolidative boost of combination therapy of patients with lung cancer. Also suggests that one of the main hazards of conventional treatment in NSCLC patients is radiation pneumonitis, triggered by radiotherapeutic intervention [88]. Cumulative proof has also been stated on the useful efficacy of certain herbal remedies i.e. Dixiong Decoction [89], Liangxue Jiedu Huoxue Decoction [90], Qingjin Runfei Decoction [91], and Shenqi Fuzheng injection [92] (Fig. 1). These formulations were demonstrated to significantly decrease the occurrence of radiation pneumonitis and progress clinical radiographic physiologic (CRP) dyspnea score and the Radiation Therapy Oncology Group (RTOG) grading score, in the allocated NSCLC patients who were under the treatment of radiotherapy. These outcomes also exposed some of the probable side effects and potential usages of precise herbal remedies in combinational treatment along with conventional chemotherapy. The extensive variety and heterogeneity of natural remedy interference and the resulting impacts still present a challenge to high-powered analysis of precise herbal remedies and their uses for evidence-based application in cancer treatment. Thus, high level quality control to confirm reliable batch preparation and systematic pharmacokinetic works are mandatory for entire trial herbal remedies as well as their activity in contradiction of lung cancer [93], not only in clinical research but also *in-vivo* rodent studies to assist evidence-based practice.

#### 3.2.3 For colon cancer

Drug interactions are common in cancer treatment because of the narrow therapeutic index and intrinsic side effects of numerous anti-cancer drugs [94]. Earlier scientific literatures stated that the activity of cytochrome P450 enzymes in the GIT wall is the utmost significant aspects which can change the bioavailability of orally consumed anti-cancer drugs that are substrates of CYP3A [95]. Herbal supplements such as Echinacea, kava, grape seed, and St John's wort (Hypericum perforatum) are also considered to be persuaders of CYP [96] (Fig. 1). Owing to the augmented practice of herbal remedies by cancer patients, more attention desires to be given to their combined use with anti-cancer drugs [97]. The consumption of St. John's wort was revealed to induce intestinal and hepatic appearance of CYP3A [98] and be beneficial for the metabolism of irinotecan [98], a camptothecin derivative which can occur in DNA injury on interaction with topoisomerase I. St. John's wort is hence used seen in the treatment of metastatic carcinoma of the colon. Earlier epidemiological studies have proved exciting patterns signifying that the treatment of herbal remedies may progress prognosis in advanced colon cancer patients when received as an adjuvant therapy [99,100]. The treatment mechanisms of TCM in cancer patients with metastasis have been conferred in terms of a hypothetical, anti-proliferation and immune-activation model of tumour development and deterioration [101].

It has been estimated that 30%–75% of colon cancer patients are using CAM, but statistical proof of survival effectiveness is still incomplete. Reported in a 10-year length clinical trial of colon cancer patients (n=193) who received Chinese medicine, in that study scientists compared the rate of survival in patients of short-term treatment of their chemotherapy with patients who continuously received long-term therapy. Moreover, they also compared the patient's survival rates who were treated with Pan-Asian drugs and vitamins (PAM + V) with the simultaneous exterior controls from the Kaiser Permanente Northern California and California Cancer Registries [102]. In this, some up-to-date techniques, such as Kaplan-Meier and traditional Cox regression were applied for scrutinizes of causal inference i.e. propensity score and marginal structural models (MSMs), that have not been previously used in studies of cancer survival patients in response to treatment with an herbal remedy. Findings advised that the combination of PAM + V with conventional treatment compared with individual conventional treatment, reduced the risk mortality rate at stage-I by 95%, stage-II by 64%, stage-III by 29%, and stage-IV by 75%. Suggests no significant variance was found between the short-term and long-term PAM + V consumption [102]. Concluded that this was a comprehensive clinical study and advocated that potential clinical trials combining PAM + V with conventional chemotherapy may be clinically justified in near future studies. Accruing clinical studies display that some TCM formulas, such as Pi-Sheng Decoction as well as Yi-Qi-Zhu-Yu Decoction, may be useful to decrease adverse effects and increasing the impact of colorectal cancer chemotherapy [103].

The Jian- Pi-Xiao-Liu Decoction, Fu-Zheng Capsule, and Qu-Xie Capsule have been applied to reduce the reappearance and metastasis of colorectal cancer in the following combination treatment after the essential incision of the patient's tumour. To ameliorate the Decoction such as QoL, Jian-Qi-Jie-Du, Jian-Pi-Yi-Qi, Fu-Pi-Yi-Wei, and Ai-Di injection has been stated to improve the anti-tumour “curative” impact of chemotherapy, diminish the adverse effects of chemotherapy, progress the function of immunity, and enhance colorectal cancer patient's survival time. Nevertheless, with the progression of colorectal cancer treatment, theories of TCM and human studies on the typing of disease variation are still falling behind. Furthermore, present studies frequently have not talked about the problems of the anti-cancer activity or the detected health benefits of TCMs therapy. It is not only needed but also really crucial for further study of the action model and the related biochemical as well as physiological mechanisms of these anti-cancer herbs, as a landmark for future TCM study [103].

#### 3.2.4 For hepatic cancer

The TCM term for unresectable hepatocellular carcinoma (UHCC) is “liver stasis” [104]. Numerous clinical trials have shown that TCM, i.e. Shentao Ruangan pills, hydroxycamptothecin, and chemotherapy can significantly decrease the symptoms, improve treatment tolerance, decline tumour size and increase body defense, decrease the degree of prevalence of side effects, and extend the survival time of UHCC patients [[105], [106], [107]].

Though the lack of quality of these studies may be disapproved singly at the international level, overall, they advocate the consumption of TCM which may permit extra trials for UHCC patients. Stated that the future clinical studies of TCM for UHCC need to have adequate procedural excellence and should be followed in agreement with the Consolidated Standards of Reporting Trials (CONSORT) declaration. Specific, thoroughly planned, multicentre, large, randomized, double-blind, controlled trials are essential [108].

#### 3.2.5 For other cancers

In the last two decades, literature has reported that Chinese herbal remedies have radio sensitization and radio-protection impacts throughout radiotherapy of cancers, i.e. bone cancer, and head and neck cancers [109]. It was proposed that combining TCM and radiotherapy not only improved beneficial outcomes but also enhanced the concert of nasopharyngeal cancer patients [110]. TCM combined with radiotherapy or chemotherapy has been shown in the literature to inhibit tumour growth, increase survival time, and improve the QoL in brain tumour metastases. The outcomes advocate that TCM is a good candidate for the treatment of a variety of cancers as shown in (Fig. 1) [111].

### 3.3 Cancer chemotherapy prevention and treatment mechanisms

#### 3.3.1 Autophagy's molecular mechanism in cancer

**Fig. 2:**
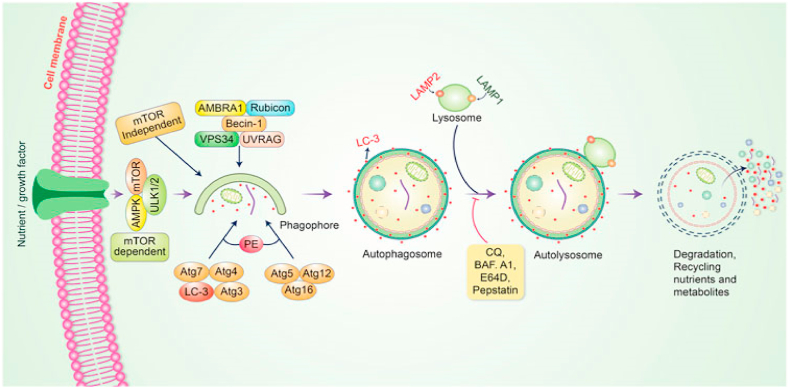
The autophagic pathway's molecular mechanism. The development of a pre-autophagosomal structure initiates autophagy. The development of pre-autophagosomal structures is aided by PI3K-AMPK and mTOR. ULK1, Vps34, and the Beclin-1 complex all plays a role in phagophore formation. Autolysosomes are formed when mature autophagosomes bind to lysosomes. Finally, acid hydrolases remove autolysosomes by producing nutrients and recycling metabolites. Reproduced with permission from [03] which was published under a CC BY license.

Autophagy is a process of cellular development that triggers the breakdown or damage of undesirable defective cellular elements by combination with lysosomes; this cellular method has been shown to perform an important job in regulating cellular functioning and homeostasis [112]. Autophagy maintains an active interlink in cell defense and a cytostatic connection in cancer-cell development [113]. Autophagy is typically started by the development of pre-autophagosomal structures documented as phagophore assembly sites (PAS) [114]. Phosphatidylinositol 3-phosphate (PI3K), an endoplasmic reticulum (ER) protein, is required for the formation of PAS [115]. The AMP-stimulated protein kinase (AMPK), mTOR, and unc-51-like autophagy activating kinase-1 (ULK1) all have been shown to support phagophore development in autophagy initiation [116], with Vps34, Vps15/p150 and Beclin-1 acting as phagophore recruiters [117]. Following the development of phagophores, phagocytosis occurs. Following that, the membrane is expanded and sealed to allow autophagosomes to form [118]. Autolysosomes are formed when mature autophagosomes bind to lysosomes [119]. Finally, autolysosomes with internal loads are spoiled by acid hydrolases and generate nutrients, while other recycling metabolites eventually maintain cellular homeostasis (Fig. 2).

#### 3.3.2 Apoptosis mechanism initiated by phytochemicals

**Fig. 3:**
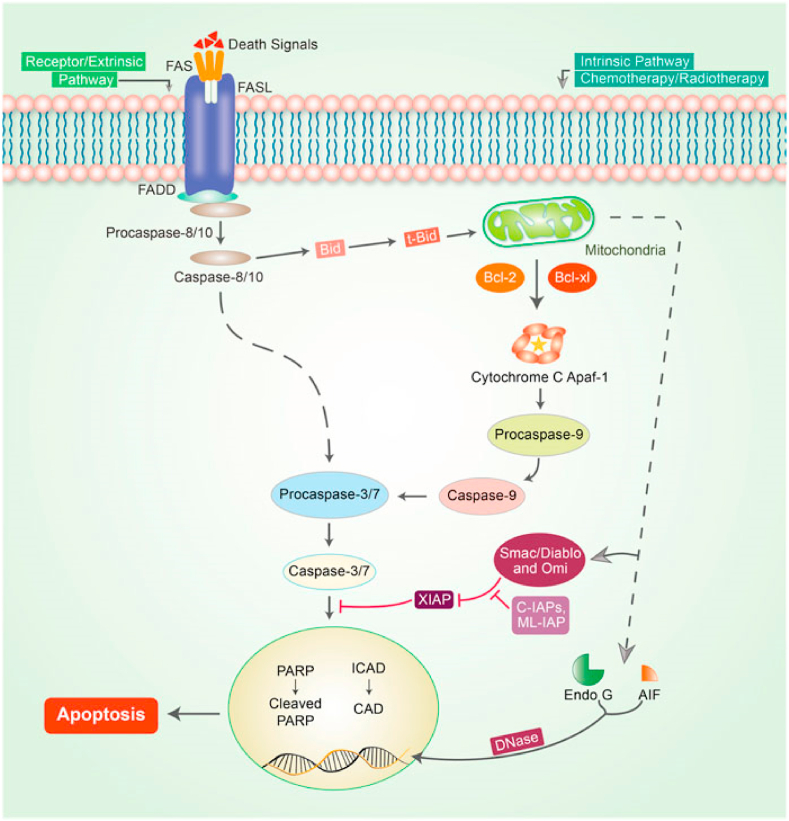
Apoptotic pathway mechanism in cancer. In this mechanism two central pathways i.e. the intrinsic and extrinsic pathways are involved to initiate apoptosis. The TNF-/TNFR1 and FasL/FasR models clearly explain the extrinsic pathway of apoptosis. An adaptor protein creates the death receptor in this case; adaptor proteins contain FADD and TRADD. The extrinsic pathway signaling causes the attachment of DRs to precise death ligands (DLs), resulting in a DISC. The complex caspase-8 activation pathway follows a pre-defined system that allows caspase-8 to separate from the DISC, regardless of whether the pro-domain of caspase-8 is kept as a part of the DISC to pledge the signaling stages of apoptosis. Nonetheless, in the majority of apoptotic cells, proteins such as caspase-9, SMAC/DIABLO, Bcl-2, Bcl-w, and MYC are usually involved in intrinsic stages. Mitochondrial deactivation is followed by loss of potential of the mitochondrial interior membrane, plenty of development of superoxide ions, decreased the development of mitochondrial biogenesis, discharge of intra-membranous proteins, and matrix calcium glutathione rupture, all of that summarize the significant potential for cancer treatment plans through activating the intrinsic phases of apoptosis in cancer cells. Caspases that initiate apoptosis, i.e. caspase-8-9, Poly (ADP-ribose polymerase (PARP), and other caspases, i.e. caspase-3-6, −7 and 10, are termed as assassin caspases (Fig. 3). Reproduced with permission from [03] which was published under a CC BY license.

Apoptosis is an important strategy for slowing the progression of cancer [120]. Because apoptosis avoidance is a cancer symbol that is unaffected by the type of cancer, focusing on apoptosis is the most effective treatment for various types of cancer. Apoptosis or programmed cell death is a normal essential pathway that is interconnected with both intrinsic as well as extrinsic pathways [121]. Nevertheless, these pathways may be intricate in the same position, which is referred to as the execution pathway [122] (Fig. 3).

It's well known that apoptosis pathways are most significant in cancer-linked treatments. Numerous phytochemical constituents were normally used as anti-inflammatory and antiviral agents. Whereas the thought of cancer mechanism expands, their antitumor properties, i.e. targeting apoptosis pathways in cancer are documented and applied [121]. Programmed cell death or apoptosis may happen in multi-cellular organisms. In cancer, insufficient apoptosis leads to unrestrained cell proliferation. Numerous signal transduction pathways are involved in the apoptosis mechanism. Apoptotic proteins can cause mitochondrial protuberance, which increases the penetrability of the mitochondrial membrane and allows apoptotic effectors to leak out [123]. The small mitochondrial-derived caspases (SMACs) are let out and then pass into the cytosol, where they bind to inhibitors of apoptosis proteins (IAPs), disable IAPs, and avert the apoptotic process from being arrested. Caspases are important in programmed cell death and are usually suppressed by IAPs, allowing the process to continue [124]. From the mitochondria, the CYT-C is let out and binds to Apaf-1 and ATP, after that it binds to pro-caspase-9 to form a protein complex apoptosome then cleaves pro-caspase to release active caspase-9, which then stimulates effecter caspase-3 [125].

The cytokine TNF is primarily induced by triggered macrophages and is the primary arbitrator of dual panoptic apoptosis. The TNF activates cell survival and inflammatory responses when it binds to its receptor. Transmembrane protein i.e. Fas ligand (FasL) is a member of the TNF family. The death-inducing signaling cascade (DISC) is formed once FasL binds to the Fas receptor (Apo-1 or CD95), which comprises the Fas-related death domain protein (FADD), caspase-8, and caspase-10 [126].

A balance between pro-apoptotic proteins i.e. BAK, BID, and BAX, and anti-apoptotic proteins like Bcl-Xl and Bcl-2 is entrenched and maintained in mammalian cells. When the mitochondrial membrane becomes permeable, caspase activators i.e. CYT C as well as SMAC are released, and proapoptotic homodimers form in the mitochondrion's outer membrane. Inhibitor caspases, such as caspases 8, 9, and 10 must bind to a specific oligomeric adaptor protein, whereas effecter caspases i.e. caspases 3, 7, and 6, are stimulated using the active initiator caspase through proteolytic cleavage as well as degradation of a variety of intracellular proteins to promote apoptosis (Fig. 3) [127] explores the apoptosis mechanisms linked to cancer and phytoconstituents.

#### 3.3.3 In various cancers, phytoconstituents modulate autophagy-apoptosis signaling

Because it eliminates damaged organs and intracellular particles and promotes lysosomal degradation, autophagy is important in cancer therapy, particularly chemotherapy. This self-digestion process protects cells from numerous intracellular and extracellular stresses while also adjusting redox balance to produce genomic and cytoplasmic stability. Earlier study reported that autophagy acts a double role in cancer, acting as both a supporter and an inhibitor of tumour growth. Nonetheless, inducing autophagy in cancer is still a viable treatment option because it induces type II apoptosis. Autophagy regulators, i.e. mTOR and AMPK, are destructively modulated during cancer initiation through tumour-suppressing factors, which cause autophagy initiation [128].

**Fig. 4:**
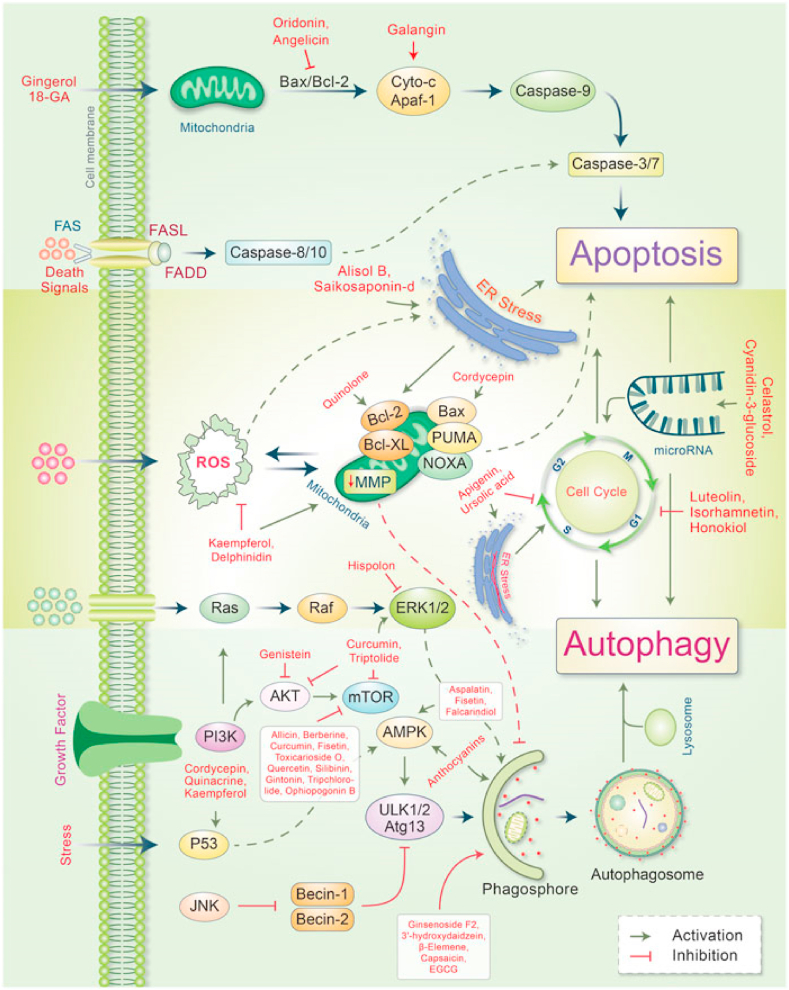
In cancer, major phytoconstituents produce signal transduction pathways that control autophagic and apoptotic cell death. Individually, phytoconstituents have been shown to stimulate the intrinsic and extrinsic apoptotic pathways by disrupting mitochondrial-caspase-9 and FAS-ligand-caspase-8 arbitrated apoptotic cell death. Phytoconstituents cause both ER stress as well as apoptotic cell death. Several phytoconstituents, however, influence mitochondrial biogenesis and confirm apoptosis-autophagic cell death. In cancer cells, phytoconstituents control the cell cycle and microRNA then causes apoptosis autophagic cell death. On the other hand, many phytochemicals stimulate autophagic signaling while inhibiting cell growth and autophagy. Reproduced with permission from [03] which was published under a CC BY license.

However, many oncogenes activate these autophagy regulators, which suppress autophagy and promote cancer development [129]. Autophagy also suppresses carcinogenesis by averting the generation of ROS, and excessive generation of ROS leads to promoting tumour generation [130]. Due to their multi-layered therapeutic properties, phytochemical compounds have been evidenced to be auspicious for the treatment of numerous cancers [131]. Reported that in some cancer patients, metabolites and synthetic products obtained from herbal components have been proven better chemopreventive impacts compared to their original compounds [132]. The evidence suggests that natural compounds selecting the autophagic-apoptotic pathways are effective cancer therapy mediators for both pathways, or that they are both dependent as well as sovereign target-specific molecular mechanisms in cancer-infected cells (Fig. 4).

Numerous phytochemical compounds and their autophagic-apoptotic impacts are presented in Table 1.

**Table 1:** Phytochemical compounds that trigger autophagy and apoptosis in numerous *in-vitro* and *in-vivo* cancer models.

Phytochemicals compounds	Concentrations	Cancer model	Molecular effects	Ref
Oridonin	8–32 μmol/L	Human hepatocellular carcinoma cell BEL-7402	Activation of caspase-3 Down-regulation of Bcl-2 and Upregulation of Bax	[133]
Norcantharidin	40 μM	Human MHCC-97H (97H) and HepG2 HCC cells	Inhibition of c-Met and mTOR	[134]
Juglanin	10 μM	Breast cancer cell MCF-7 and SKBR3	Regulation of ROS and JNK	[135]
Isoliquiritigenin	25 μM	Human ovarian cancer cell, OVCAR5 and ES-2	Cleaved caspase-3, augmented LC3BII, and Beclin-1 level	[136]
Cucurbitacin B	200 μM	Breast cancer cell MCF-7	Elevation of γH2AX, phosphorylation of ATM/ATR, ROS	[137,138]
Kaempferol	50 or 100 μM	Colorectal cancer cell HCT116, HCT15, and SW480	Produced ROS and p53 signal	[139]
Carnosol	25 μM	Human breast cancer cell MDA-MB-231	Raise p21/WAF1 and downregulate p27	[140]
Ursolic acid	10–40 μM	Prostate cancer cell PC3	Augment Beclin-1/Atg5 and inhibits Akt/mTOR	[141]
Triptolide	200 nM	Human pancreatic cancer cell S2-013, S2-VP10, and Hs766T	Inhibits of Akt-mTOR-P70S6K	[142]
Luteolin	100 μM	Human liver cancer SMMC-7721	Augment appearance of caspase-8, decline bcl-2	[143]
α-Mangostin	5–10 μM	Human brain cancer cell, GBM8401 and DBTRG05MG	Activation of AMPK	[144]
Quercetin	15 μM	Lymphoma cell BC3, BCBL1 and BC1	Inhibits PI3K/Akt/mTOR and Wnt/β-catenin	[145]
γ-tocotrienol	10 μmol/L	Breast cancer cell MCF-7 and MDA-MB-231	Activate AMPK, down regulate Ang-1/Tie-2	[146]
Thymoquinone	40–60 μM	Oral cancer cell SASVO3, SCC-4, OCT, SAS	Augment appearance of LC3-II and Bax	[147]
*N*-desmethyldauricine	150 μM	Lung cancer cell H1299	Inhibition of Ulk-1/PERK/AMPK/mTOR	[148]
Quinacrine	15 μM	Colon cancer cell HCT-116/HCT-116/HCT-116	Activation of p53, p21, and inhibition of topoisomerase	[149]
Chloroquine	50 μM	Pancreatic cancer cell MiaPaCa2 and S2VP10	Diminished the level of O2	[150]
Tangeritin	10 μM	Breast cancer cell MCF7, MDA–MB–468 and MCF10A	Induce CYP1 and CYP1A1/CYP1B1 protein expression	[151]
Myricetin	100 μM/L	Prostate cancer cell PC3, DU145	Knockdown the interaction between P1M1/CXCR4	[152]
Hesperetin	350 μM	Lung cancer cell H522	Knockdown caspase-3/9, p53, Bax Upregulate Fas, FADD and caspase-8	[153]
Delphinidin	80 μM	Breast cancer cell MDA-MB-453 and BT474	Suppression of mTOR Activation of the AMPK	[154]
Epigallocatechingallate (EGCG)	500 μM	Human glioblastoma cell T98G and U87MG	Raise the level of ROS	[155]
Epicatechin-3-O-gallate (ECG)	36 μM	Prostate cancer cell LNCaP and PC-3	Reduced the progression of carcinofenic cell	[156, 157]
Cyanidin-3-glucoside (C3G)	20 μM	Human breast cancer cell MDA-MB- 231 and Hs-578T	Inhibiting STAT3/VEGF and miR124 mediated downregulation STAT3	[158]
Benzyl isothiocyanate (BITC)	6.5 μM	Pancreatic cell BxPC-3 and PanC-1	Decline the phosphorylation of PI3K/Akt/FOXO1/PDK1/mTOR/FOXO3a	[159]
Phenethyl isothiocyanates (PEITC)	10 μM	Breast cancer cell MDA-MB-231 and MCF-7	Decrease the expression of HER2, EGFR and STAT3	[160]
Piperlongumine (PL)	6 μM	Lung cancer cell A549 and A549/DTX	Control PI3K/Akt/mTOR	[161,162]
Saikosaponin-d	10 μM	Breast cancer cell HeLa and MCF-7	Calcium mobilization, induce CaMKKβ-AMPK-mTOR	[163,164]
Guttiferone K	20 μM	Human HCCs HuH7 and HepG2	Decrease phosphorylation of Akt /mTOR, raise ROS	[165,166]
Licochalcone A	20 or 50 μM	Breast cancer cell MCF-7	Suppression of PI3K/Akt/mTOR pathway	[167]
Ophiopogonin B	10 μM	Lung cancer (NSCLC) cell NCI–H157 and NCI–H460	Inhibition of PI3K, Akt, mTOR	[168]
Berberine	100 nM	Human glioma cell U251 and U87 GBM	Inhibition of AMPK/mTOR/ULK1	[169]
Capsaicin	150 μM	Human nasopharyngeal carcinoma cell NPC-TW01	Downstream of PI3K/Akt/mTOR, increase caspase-3 activity	[170]
Celastrol	1.5 μM	Human prostate cancer cell LNCaP, 22Rv1, DU145 and PC-3	Upstream of miR-101	[171]
Curcumin	25 μM	Malignant mesotheloma cancer cell MM-B1, H-Meso-1, and MM-F1	Raise Bax/bcl-2 ratio, p53 expression, activation of caspase 9, cleavage of PARP-1	[172]
Epigallocatechin gallate (EGCG)	100 nM	Vascular endothelial cell U-937	Decrease TNF-α, inhibit VCAM1, LC3A, LC3B	[173]
Evodiamine	10 μM	Gastric cancer cell SGC-7901	Activates beclin-2, Bax, downregulates Bcl-2	[174]
Genistein	50–100 μM	Ovarian cancer cell A2780	Diminish Akt/mTOR phosphorylation	[175]
Gingerol	300 μM	Human colon cancer cell SW-480 and HCT116	Inhibition of JNK, ERK1-2, and P38 MAPK	[176]
Ginsenoside F2	100 μM	Breast cancer cell MCF-7	Elevated Atg-7 Cleaved PARP	[177]
Oleanolic acid	100 μg/mL	Human pancreatic cancer cell Panc-28	Modulate JNK and mTOR pathway	[178]
Honokiol	40 μM	Human glioblastoma cell LN229, GBM8401 and U373	Decrease p-PI3K, p-Akt and Ki67	[179]
Magnolol	40 μM	Human glioblastoma cell LN229, GBM8401 and U373	Decrease p-PI3K, p-Akt and Ki67	[179]
Alisol B	30 μM	Breast cancer cell MCF-7, SK-BR-3, and HeLa	Activation of Ca^2+^/AMPK/Mtor	[180]

#### 3.3.4 ATP-dependent chromatin remodeling

Chromatin remodeling refers to the transformation of nucleosomes on DNA by enzymes. It is well understood that chromatin is a shortened as well as frequently unreachable shape in which histone and non-histone proteins wrap genomic DNA. When DNA is damaged, effective and precise repair of the genetic material confirms its stability and prevents the development of damage that could cause cell death [181]. By using post-translation histone alterations and ATP-dependent chromatin remodeling, cells can control the structure of chromatin and rise the availability of overhaul machinery to scratches ingrained in chromatin [182].

To repair DNA, chromatin remodeling disrupts DNA links by using the energy of ATP, transferring and eliminating nucleosomes [183]. Numerous chromatin remodeling multiplexes are formed during the process, including INO80 chromatin remodeling aspects composed of INO80 ATPase or associated SWR1-like factors, namely the p400 ATPase, which has an extended inset in the center of the preserved ATPase realm, and CHD family members [184]. The authors [185] reviewed the genetics, genomics, and mechanisms of ATP-dependent chromatin remodeling. Because some cancer cells have flaws in single or more characteristics of the DNA damage response (DDR), these cancer cells are more susceptible to cancer treatments that aim to direct the tumour-linked DDR defects [186].

#### 3.3.5 Epigenetics and DNA methylation

Methylation of DNA is an inherited process that occurs during cell division. DNA methylation occurs most commonly in adult somatic tissue at CpG sites, where a cytosine and a guanine are separated by a phosphate. According to one study, between 60% and 90% of all CpGs in mammals are methylated [187]. Unmethylated CpGs can be found in the 5′ regulatory areas of various genes. During the development of cancer, gene promoter CpG groups undergo anomalous hypermethylation, which causes transcriptional quieting and is inherited by daughter cells after cell division [188].

#### 3.3.6 microRNAs (miRNA)

In recent years, MiRNAs have received increased focus in cancer research, and their regulation by herbal compounds is at an early stage in both chemotherapy research [189]. MiRNAs post-transcriptionally control the expression of the gene by targeting the 3′ untranslated domains of accurate messenger RNAs for degradation [190]. MiRNAs function as post-transcriptional controllers by interacting with reciprocal orders on single or more messenger RNA transcripts [191].

MiRNA can be completely or moderately balanced to the miRNA target in animals, allowing a single miRNA to target multiple locations on the same or different mRNAs [192]. MiRNA seems to bind in mRNA beforehand it can be translated into proteins that turn on and off of genes [193].

The dicer enzyme converts the pre-miR to the double-stranded 22-nucleotide miRNA. The duplex is then unraveled into two strands: the degraded passenger strand and the guide strand, both of that are combined with the RNA-induced silencing complex (RISC). Based on complementarity, RISC combined with miRNA can bind to the 3′ untranslated regions (UTR) of target mRNAs, creating a block in the translation or degradation of mRNA [194]. While miRNAs play a vital role in modulating cellular differentiation and proliferation, their dysregulation has been connected to cancer, and they can act as a tumour suppressor and persuader oncogenes. According to reports, the deficiencies of MiRNA have been linked to various diseases including cancer. Excess c-Myc, a protein found in many cancers in mutated forms, advocates that miRNA plays a vital function in the formation of cancer [195].

#### 3.3.7 NF-κB pathway

Cancer progression is linked to the NF-κB. The NF-κB transcription factors are fast-acting transcription factors that are inactive in cells and do not necessitate new protein synthesis for the activation, unlike c-Jun and STATs. As a result, NF-κB can be a primary responder to potentially injurious cellular stimuli. ROS, IL-1, TNF, and lipopolysaccharide are all NF-κB inducers (LPS). A family of IBs, whose ankyrin repeat domains mask the NF-κB nuclear localization signals, captures the NF–B regulators in the cytoplasm (NLS). When stimulated, IBs are ubiquitinated and degraded by IB kinases (IKK). Then NF-κB gets enters the nucleus and activate the appearance of precise genes with NF-κB DNA-binding sites nearby. The NF-κB continues to express its repressor, IB, which reinhibits NF-κB and forms an auto feedback loop, resulting in oscillating NF-κB activity] [196]. In tumour cells, NF-κB is stimulated, and hindering NF-κB can induce tumour cells to avert proliferating, die, or become highly sensitive to the action of anti-tumour drugs [197].

#### 3.3.8 Phytochemical chemoprevention mechanisms

**Fig. 5:**
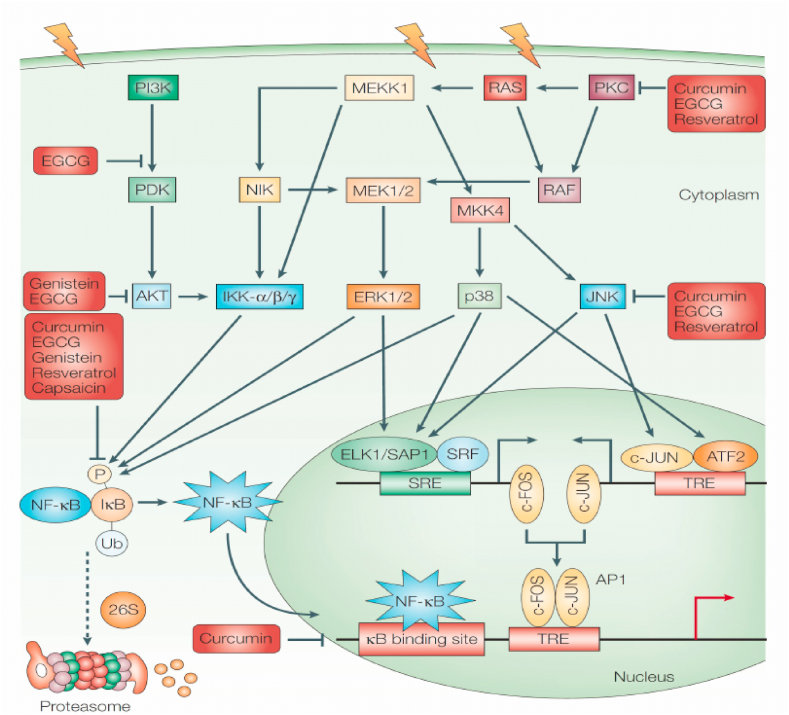
Phytochemical mechanism of action of chemopreventive agents on NF-kB and AP1. Reproduced with permission from Ref. [201].

Inactivation of the MAPK pathways, PKC, and P13k pathways causes uncontrollable cell growth and the transformation of healthy cells into cancer cells. A variety of phytochemical constituents have been identified as chemo preventive agents capable of monitoring these enzymes and preventing abnormal cell growth and proliferation [198]. NF-kB can be activated and released by inflammatory, ROS, cytokines, and cancer-inducing agents. After being released the NF-kB is relocated to the nucleus. The NF-kB binds to and exhibits genes in the nucleus which prevent healthy cells from dying and cause cancer cell multiplications, invasion, inflammation, and metastasis [199] (Fig. 5).

Activator protein 1 (AP 1) is a heterogeneous set of dimeric proteins which are caused by TNF and IL-1 as well as ecological stress. Its activation is linked to cell progression, inflammation, and cell damage regulation. According to scientific literature, it regulates genes involved in apoptosis, cell adjustment, integration, and multiplication, as well as cancer and tumour development [200]. NF-kB and AP1 are the primary sites of various phytochemical chemo preventive agents i.e. epigallocatechin gallate, curcumin, capsaicin, gingerol, genistein, and resveratrol (Fig. 5).

#### 3.3.9 Nrf2 pathway

The Nrf2 is a transcription factor that regulates antioxidant responses [202]. As a result, cancer patients may experience oxidative stress. The Nrf2 pathway is critical in cancer chemotherapy prevention and treatment research. Nrf2 is also a leucine zipper (bZIP) transcription factor which is separate from the JUN and FOS bZIP families [203]. The Keap1 binds to Nrf2 in the cytoplasm in the absence of stress. When critical cysteine residues in Keap1 are interrupted by oxidative or other electrophonic stress, Nrf2 can translocate into the nucleus [204].

**Fig. 6:**
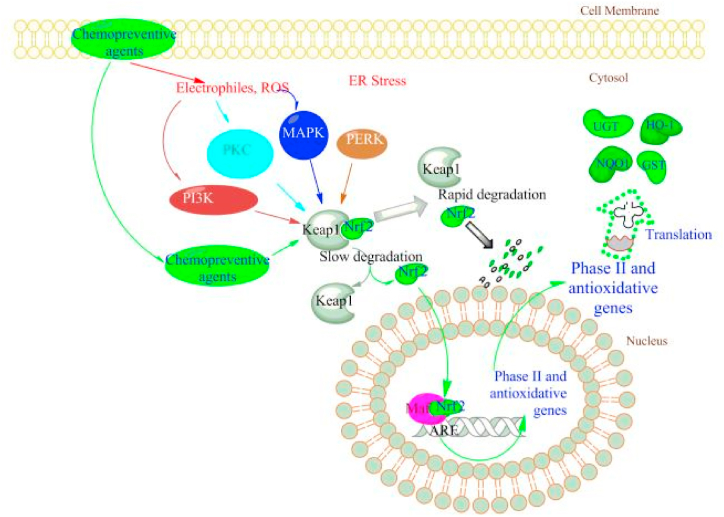
Phytochemicals regulate Nrf2-mediated gene transcription. Keap1 protein keeps Nrf2 in the cytoplasm under homeostatic conditions. Chemopreventive phytoconstituents directly interact with Keap1 cysteine residues, causing Nrf2 to be released from the complex. Chemopreventive drug-produced ROS can stimulate an extensive range of kinase signaling pathways, comprising PI3K, PKC, and MAPK, and all can cause Nrf2 expression and translocation from the cytosol to the nucleus. Reproduced with permission from Ref. [206] which was published under a CC BY license.

In the advocate area of numerous anti-oxidative genes, the Nrf2 gets a heterodimer structure with slight Maf proteins and then binds to the Antioxidant Response Element (ARE), pledging transcription [205]. Chemopreventive drugs cause Keap1 to dissociate from Nrf2, resulting in the induction of Phase II genes, which are then translated into proteins for chemoprevention impacts. The Nrf2 has received a lot of attention, and earlier literature by Keum provided up-to-date information on this signal pathway [206] (Fig. 6).

#### 3.3.10 PI3K signal pathway

The PI3Ks regulate cell growth, proliferation, differentiation, survival, and intracellular trafficking. They act as intracellular signal transducer enzymes by phosphorylating the 3-position –OH group of the phosphatidylinositol inositol ring (Ptdlns) [207]. Stimulated PI3K induces both Ptdlns(3,4,5)P3 as well as Ptdlns(3,4)P2, which are bound by AKT. The translocation of AKT to the plasma membrane is restricted owing to the Ptdlns (3,4,5) P3 and Ptdlns(3,4)P2. Similarly, the pleckstrin homology domain of phosphoinositide-dependent protein kinase 1 (PDK1) binds to and translocates to the plasma membrane with Ptdlns(3,4,5)P3 and Ptdlns(3,4)P2. Because stimulated PDK1 and AKT colocalize, AKT is phosphorylated on threonine 308 by PDK1, resulting in partial AKT activation. The AKT gets completely stimulated when serine 473 is phosphorylated by the TORC2 complex of the mTOR protein kinase. As a result, PI3K activity is crucial to cellular transformation and cancer progression. Suggests that inhibiting PI3K becomes a treatment plan for cancer progression [208].

#### 3.3.11 PI3K/AKT/mTOR signaling pathway

**Fig. 7:**
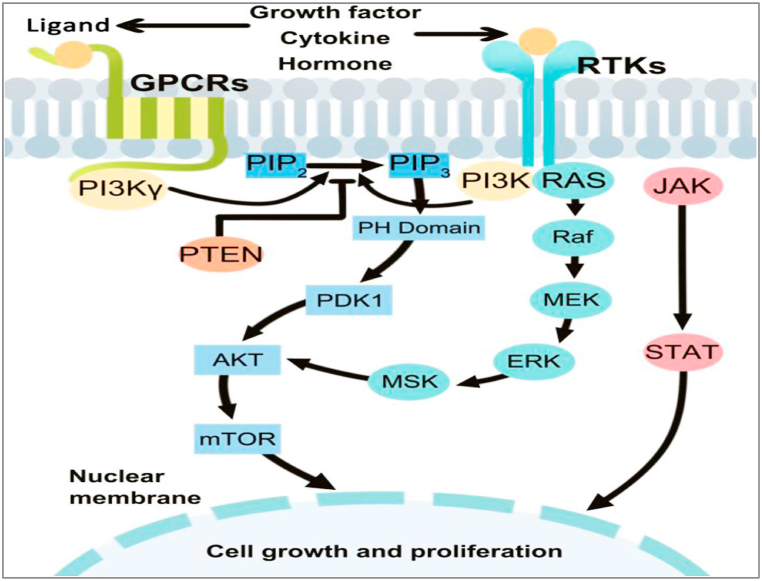
A summary of the PI3K/AKT/mTOR signaling pathway. Reproduced with permission from Ref. [214] which was published under a CC BY license.

The PI3K is a lipid kinase cluster that creates 3’ subunits such as p85 regulatory, p55 regulatory, and p110 catalytic [209]. Based on the structural differences and specific substrates PI3K is divided into three classes [210]. Class I PI3Ks were further subdivided into IA and IB. Class IA PI3K, a heterodimer of the p58 regulatory subunit and the p110 catalytic subunit are utmost implicated in human cancer [211]. Class IA PI3K comprises p110, p110, and p110 catalytic subunits derived from several genes i.e. PIK3CA, PIK3CB, and PIK3CD, respectively, whereas class IB PI3K contains only p110 derived from PIK3CG [212]. The p85 regulatory subunit is made up of three parts: p85a, p85b, and p55g, each of that are encrypted by a different gene, including PIK3R1, PIK3R2, and PIK3R3 [213]. The p85 regulatory subunit binds and assimilates signals from a broad range of transmembrane and intracellular proteins [214] (Fig. 7).

#### 3.3.12 Plk1 expression

Plk1 is an enzyme that contains 603 amino acids. Furthermore, the *N*-terminus kinase area and two preserved polo-box areas of 30 amino acids at the *C*-terminus. Kinase activity is controlled by the polo-boxes which are functionally significant for both auto-inhibition and subcellular localization [215,216]. During the G2/M transition, Plk1 is the first gene to be activated. Because it is overexpressed in tumour cells, it is referred to as a proto-oncogene. Plk1 is thought to be involved in cell cycle development, which has an oncological significance. Plk1 overexpression has been discovered in cancer-infected nude mouse cells [217]. Plk1 is involved in tumour suppressor pathways associated with p53 [218]. An earlier review discussed Plk1, a critical regulator of mitosis, and its promising role in non-small cell lung cancer treatment [219].

#### 3.3.13 Inhibition of tumour angiogenesis

**Fig. 8:**
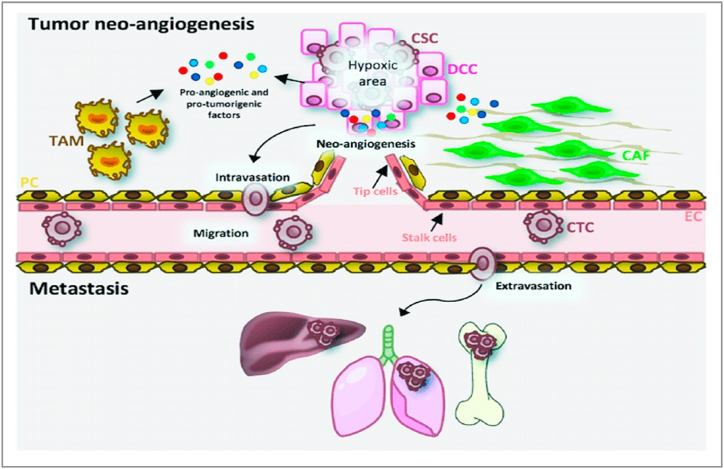
Angiogenesis of the tumour and the metastatic process. The proteins such as VEGF-A, MMPs, HIF-1A, chemokines, cytokines, and growth factors are among the proangiogenic and protumorigenic factors released by cancer cells. Endothelial cells (ECs) in tip cells, which direct emergent vessels, and stem cells, which are involved in vessel stability, are stimulated by VEGF-A. Furthermore, cancer cells secrete cytokines that stimulate cancer-related fibroblasts (CAFs) and stimulated tumour-related macrophages (TAMs), promoting cancer stem cell intravasation (CSCs). Cancer cells circulating in the bloodstream arrive in the target organ, appear, and begin to proliferate and spread. Reproduced with permission from Ref. [220] which was published under a CC BY license.

Angiogenesis is the physiological process that can develop new blood vessels to collect nutrients. It is the primary stage in the development of cancer from latent to cancerous, which leads to the usage of inhibitors in angiogenesis. Tumours cause new blood vessel growth by releasing a variety of growth factors, including VEGF, which causes blood capillaries to form within the tumour. Protein kinase G (PKG) regulates beta-catenin levels in healthy cells, promoting angiogenesis. Angiogenesis is also needed for tumour metastasis to spread. Thus, the use of specific ingredients inhibits the development of new blood vessels, which may assist to fight tumours, as they require a lot of oxygen as well as nutrients to grow. The FGF family is primarily composed of single-chain peptides [220] (Fig. 8).

The FGF-1 promotes the proliferation and differentiation of all cells, including endothelial as well as smooth muscle cells, which are required for arterial vessel formation, whereas VEGF promotes the development of new capillaries [221]. Binding to the VEGFR-2 starts a tyrosine kinase signaling cascade that increases vessel permeability by inducing NO, proliferation, and relocation. In healthy cells, protein kinase G (PKG), an *anti*-VEGF enzyme, limits beta-catenin, which promotes angiogenesis. Cancer cells were discovered to stop producing PKG.

**Fig. 9:**
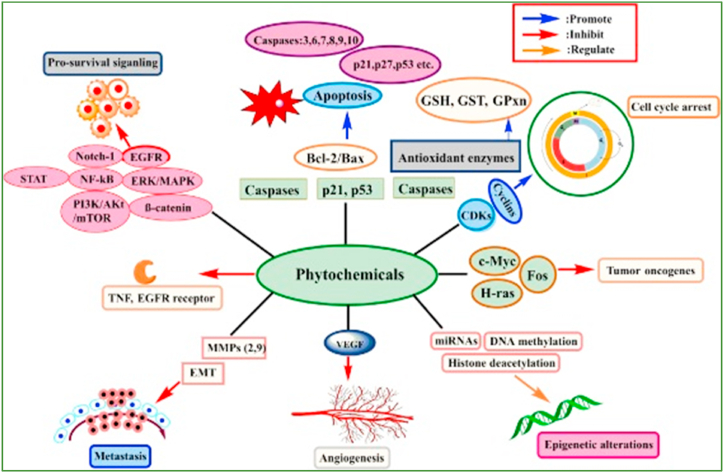
Various phytochemicals are involved in regulating an extensive range of molecular processes in order to modulate skin cancer. Reproduced with permission from Ref. [222] which was published under a CC BY license.

Phytochemical constituents can inhibit the proliferation of skin cancer by inducing apoptosis and decreasing the appearance of anti-apoptotic factors such as Bcl-XL and X-IAP, and controlling iNOS and COX-2. Herbal remedies can disrupt skin cancer cells by preventing angiogenesis and metastasis, arresting the cell cycle, suppressing EMT, regulating epigenetic modification, and decreasing MMP and COX-2 enzyme control (Fig. 9) [222].

#### 3.3.14 Anticancer phytochemical compounds and their molecular mechanism of CD-induced tumour cell death

Several cytotoxic phytochemical compounds are summarized in Table 2. Compounds exhibiting cytotoxic/anticancer effects, which are investigated through the use of survival tests such as MTT and/or CCK-8 which exhibits that IC_50_ values are in the range of nano-molar to micro-molar.

**Table 2:** Bioactive compounds showed Pharmacological activities and their IC_50_ values.

Class Category	Name of Compound	Pharmacological activities described in references	Concentration IC_50_/EC_50_/minimal inhibitory concentration (MIC)	*In-vitro/In-vivo* Model
Aporphine	(−)-Anonaine	Cytotoxicity [223]	8.6–28.9 μM	AGS, DLD1, HA59T, and HepG2
	Bidebiline E	Anti-bacterial [224]	6.25 μg/mL	Mycobacterium tuberculosis
		Inhibition of wnt protein [225]	20.2 μM	SW480
Proaporphine	(+)-Stepharine	Cytotoxicity [179]	9.4–9.9 μg/mL	MCF-7, MDA-MB-231
Oxoaporphine	Liriodenine	Cytotoxicity [226]	0.57–2.33 μg/mL	KB, A549, HCT-8, P-388, and L-1210
	Lanuginosine (oxoxylopine)	Cytotoxicity [227]	1 μg/mL	Unavailable
	Oxostephanosine	Cytotoxicity [227]	1 μg/mL	Unavailable
	Oxostephanine	Cytotoxicity [228]	1.47–1.73 μg/mL	SPC-A-1 and BEL-7402
Azafluorene	6,8-Dihydroxy-7-methoxy-1-methylazafluorenone	Cytotoxicity [229]	2.64–3.58 μg/mL	A549, GLC4, and adrinamycin-resistance GLC4
		Apoptosis [230]	20–55 μM	HL-60, U937, MOLT-4, MDA-MB-231, and HepG2
	Polylongine	Cytotoxicity [226]	9.94–10.41 μg/mL	MCF-7 and MDA-MB-231
Anthraquinones	Marcanine A	Cytotoxicity [231]	1.53–11.78 μM	BEL-7402, K562, SPCA-1, and SGC-7409
Prenylated Benzopyran	Polycerasoidol	Anti-inflammatory [232]	4.9 μM	Inhibition of mononuclear leukocyte adhesion to endothelium
	1-(2-furyl) pentacosa-16,18-diyne	Anti-viral [233]	43.3 μg/mL	DTat/RevMC99 syncytium assay for HIV
	23-(2-furyl)tricosa-5,7-diynoic acid	Anti-viral [233]	8.9 μg/mL	Same as above
Acetogenin	Debilisone E	Cytotoxicity [234]	18.4–40.3 μg/mL	HepG2, A549, HCC-S102, HL-60, and P-388
Tetrahydroproto berberine	(−)-stepholidine	Cytotoxicity [226]	16.56 μg/mL	MCF-7
Amides	*N*-trans-Feruloyltyramine	Cytotoxicity [226]	21.17–25.54 μg/mL	MCF-7, MDA-MB-231, HepG2, Hep3B
	*N*-trans-p-Coumaroyltyramine	Cytotoxicity [226]	17.35 μg/mL	MCF-7
Sesquiterpenes	Polyalone A	Cytotoxicity [235]	18.9–24.8 μM	HeLa, A549, MCF-7, and HL-60
	9-Ketocyclocolorenone	Cytotoxicity [235]	20.5–26.2 μM	Same as above
	Blumenol A	Cytotoxicity [235]	24.5–28.2 μM	
	(−)-Methyl dihydrophaseate	Cytotoxicity [235]	22.6–27.1 μM	
	Bis-enone	Cytotoxicity [235]	25.6–30.1 μM	
Triterpene	Suberosol	Cytotoxicity [236]	34.30 μg/mL	SPC-A-1
	24-Methylenecycloartane-3, 16 , 23-triol (longitriol)	Cytotoxicity [237]	40.3 μM	MRC-5
Flavonoids	Quercetin	Anti-oxidant [238]	1.56 μg/mL	Trolox equivalent antioxidant capacity (TEAC) assay
	Quercetin-3-*O*-glucopyranoside	Anti-oxidant [238]	1.56 μg/mL	TEAC assay
	Rutin	Anti-oxidant [238]	1.56 μg/mL	TEAC assay
8-Oxoprotoberberine	(−)-8-oxo-2,9,10-Trihydroxy-3-methoxyberberine (consanguine B)	Cytotoxicity [239]	24.1 μM	MCF-7

#### 3.3.15 Oxidative stress

##### 3.3.15.1 Mechanism of oxidative stress-related carcinogenesis

Oxidative stress has been connected to cellular stress in the development of cancer. Reactive oxygen species (ROS) contribute to tumorigenesis through several mechanisms, such as DNA damage, inflammation, immune dodging, signaling pathway regulation controlling autophagy and apoptosis, angiogenesis, and drug resistance [240]. Tumorigenesis has also been connected to free radicals which cause chromosomal aberrations and oncogene instigation [241].

The impact of ROS on cancer varies according to organ type and tumour grade. Increased ROS levels normally cause cell death, but cancer cells avoid this by activating dozens of new oncogenes. ROS such as hydrogen peroxide, superoxide, and hydroxyl free radicals, are mostly formed during the consumption of oxygen metabolic reactions [242].

Moderate increases in ROS contribute to several pathologic conditions, among which are a proliferation of cancer cells and the formation of metastatic colonies [243]. ROS triggers diverse signaling pathways involving mitogen-activated protein kinase/extracellular signal-regulated protein kinases 1/2 (MAPK/ERK1/2), p38, JNK, and PI3K/AKT. These, in turn, activate the NF-κB, MMP, and VEGF [244].
On the other hand, a high level of ROS can be reached by numerous anti-cancer treatments, and suppress tumour metastasis by killing cancer cells owing to the oxidative nature of the molecules. Henceforth, there are two ROS targeting strategies for hindering tumour angiogenesis and metastasis, to either increase tumorigenesis or lead to apoptosis [245]. Phytocompound such as Nimbolide obtained from Azadirachta Indica produce excessive ROS generation, thus inhibiting proliferation and metastasis via mitochondrial-mediated apoptotic cell death [246].

##### 3.3.15.2 Role of phytoconstituents in the treatment of cancer based on molecular pathway

⁃ **MAPK Pathways:** Phytochemicals can target the extracellular signal regulated kinase (ERK) and mitogen-activated protein kinase (MAPK) pathway, which essentially regulates cellular growth and survival. These plant compounds potentially control cancer development through various mechanisms [247]. The phytocompounds such as ursolic acid, kaempferol, resveratrol, gingerol, sulforaphane, genistein, and isothiocyanates were reported to cause cancer cell apoptosis through MAPK and ERK pathways [248].
⁃ **Akt Signaling Pathways:** In cancer control and development, Akt/PI3 signaling pathway plays a crucial role. Levels of epidermal growth factor (EGF) regulate a series of molecular mechanisms including activation of NF-κB and phosphorylation of Akt leading to resistance to apoptosis and uncontrolled cell proliferation, while downstream, it leads to the regulation of caspases, Bcl-2, and glycogen synthase kinase 3-β (GSK3β), and mammalian target of rapamycin (mTOR) [249]. Alkaloids and phenolics compounds are significantly contributed to controlling the expression of these factors. Resveratrol, curcumin, luteolin, flavone, and sulforaphane showed anticancer properties via cell cycle arrest and apoptosis, hindering Akt/PI3K signaling, proapoptosis, antiproliferation and anti-invasion [250].
⁃ **JAK/STAT Signaling Pathways:** Phytochemical compounds significantly lead to induce cell death in several cancer forms by deterring the activity of JAK/STAT signaling and activating apoptotic cascades [251]. Curcumin, resveratrol and EGCG, inhibit the translocation and gathering of β-catenin in the nucleus by stimulation of glycogen synthase kinase 3 (GSK3) [252].

## 4 Conclusion and future outlooks

The global prevalence of cancer continuously rising, and new methods are being developed to confirm this lethal ailment is accomplished therapeutically. The main problem to emerging specific anticancer agents is the complexities of cancer pathobiology. Autophagy and apoptosis are two distinct essential cellular pathways involved in the progression and regulation of cancer. Due to the deficiencies in apoptosis signaling pathways, various kinds of cancer are becoming resistant to chemotherapy. Autophagy may be considered a distinct cell fate mechanism for the development of specific anticancer agents. Further in-vitro and in-vivo research is needed to well understand cancer pathobiology, allowing the complete potential of autophagy and apoptosis selected drug design to be used.

People are getting interested gradually to use herbal remedies as fruitful sources for cancer treatments. Surely, there is ever-increasing evidence that herbal compounds with anticancer activity can modulate a variety of signaling pathways comprising autophagy and apoptosis pathways. The anticancer impacts of phytoconstituents exhibited to be selective and precise to cancer-infected cells, involving autophagy and apoptosis initiation. As a result, a wide range of phytoconstituents is beneficial sources of anticancer agents. Curcumin, resveratrol, EGCG, and berberine are some of the most well-known phytoconstituents that have been shown in in-vitro and in-vivo studies to have anticancer activity by modulating the autophagy and apoptosis pathways in several types of cancer. Because autophagy is context-dependent in cancer patients, targeting this important cellular pathway may not constantly be advantageous. Moreover, many herbal constituents target several signaling pathways which may be shared by several cellular structures, making the development of phytochemical-based anticancer agents challenging. Furthermore, combined system pharmacology and computational methods can be used to well-known phytochemical constituents that have anticancer effects.

Although the clinical use of herbal medicines is limited due to their poor bioavailability, advances can be made by using a drug delivery system based on nanotechnology. The potential and challenges of phytochemical-intervened autophagy and apoptosis targeting, according to the high points of this review, could solve new methods and plans for the development of novel chemotherapy to treat a variety of cancers. Finally, future challenges and likely outlooks have been presented in the hope of enhancing anticancer efficiency and accelerating the translational development of specific nanomedicine or nanotechnology for selected cancer treatment based on the autophagy-apoptosis pathway. Nanoparticle-based drug delivery systems (NDDSs) have been widely used in cancer diagnosis, treatment, and imaging due to their exceptional cancer-targeting efficacy and low toxic properties. The NDDSs, however, are now being challenged by exceptional researchers due to flaws such as poor patient prognosis, increased erraticness, and multidrug resistance (MDR).

Combined targets of nanoscience along with naturally derived bioactive components are very attractive and have recently been developed in the combination formula with standard drugs to improve clinical outcomes. Hence, it is instantly required to essential with designing new therapeutical lines to study detailed the initial diagnosis as well as the pathogenesis of cancer thus targeting phytocompounds through the autophagy-apoptosis pathway.

## Declarations

### Author contribution statement

All authors listed have significantly contributed to the development and the writing of this article.

### Funding statement

This research did not receive any specific grant from funding agencies in the public, commercial, or not-for-profit sectors.

### Data availability statement

Data will be made available on request.

### Declaration of interest's statement

The authors declare no conflict of interest.
